# Pooling *Operations in Deep* Learning: From “Invariable” to “Variable”

**DOI:** 10.1155/2022/4067581

**Published:** 2022-06-20

**Authors:** Zhou Tao, Chang XiaoYu, Lu HuiLing, Ye XinYu, Liu YunCan, Zheng XiaoMin

**Affiliations:** ^1^School of Computer Science and Engineering, North Minzu University, Yinchuan 750021, China; ^2^Key Laboratory of Image and Graphics Intelligent Processing of State Ethnic Affairs Commission, North Minzu University, Yinchuan 750021, China; ^3^School of Science, Ningxia Medical University, Yinchuan 750004, China; ^4^Research Institute for Reproductive Medicine and Genetic Diseases, Wuxi Maternity and Child Health Hospital, Wuxi 214002, China

## Abstract

Deep learning has become a research hotspot in multimedia, especially in the field of image processing. Pooling operation is an important operation in deep learning. Pooling operation can reduce the feature dimension, the number of parameters, the complexity of computation, and the complexity of time. With the development of deep learning models, pooling operation has made great progress. The main contributions of this paper on pooling operation are as follows: firstly, the steps of the pooling operation are summarized as the pooling domain, pooling kernel, step size, activation value, and response value. Secondly, the expression form of pooling operation is standardized. From the perspective of “invariable” to “variable,” this paper analyzes the pooling domain and pooling kernel in the pooling operation. Pooling operation can be classified into four categories: invariable of pooling domain, variable of pooling domain, variable of pooling kernel, and the pooling of invariable “+” variable. Finally, the four types of pooling operation are summarized and discussed with their advantages and disadvantages. There is great significance to the research of pooling operations and the iterative updating of deep learning models.

## 1. Introduction

Deep learning was proposed by Hinton [1] in 2006. A Convolutional Neural Network (CNN) was proposed by Le et al. [2], which is the first real multilayer structure learning algorithm. Deep learning has been widely used in various fields of multimedia such as text, image, and video, and it has become a research hotspot in the field of images. Convolution operation is the main step of the CNN, but it makes the whole network have a large number of calculations [3]. And it takes a long time for network training. Therefore, the pooling operation is an essential step in CNN.

Pooling operation is a common step in CNN and is also called subsampling operation or downsampling operation. When CNN was constructed, the pooling operation is usually placed behind the convolution layer. From the perspective of lightweight, pooling operation can reduce feature dimensionality, computation complexes, parameter number, and memory consumption and suppress noise. From the perspective of improving image quality, pooling operation can improve the horizontal movement invariance, scale invariance, and rotation invariance of the model. The pooling operation improves the robustness to image bias and rotation. From the perspective of the deep learning model, pooling operation can prevent overfitting. Pooling operation can improve the generalization ability of the model and expand the receptive field. Hence, it is very interesting to discuss pooling operation.

## 2. The “Variable” and “Invariable” of Pooling Operation

**Figure 1:**
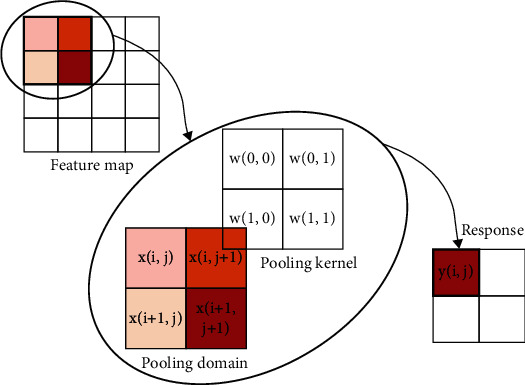
The process of pooling operation.

There are five parts in pooling operation, such as pooling domain, pooling kernel, step length, activation value, and response value [4]. The process of the pooling operation is shown in Figure 1. The “invariable” of pooling operation means that there is no change in the shape and size of the pooling domain and the shape, size, and parameter number of the pooling kernel. The “variable” of pooling operation means that there are two types: first is pooling domain changing and second is pooling kernel changing. About pooling domain changing, the shape and size are changed in the pooling domain. About pooling kernel changing, the shape, size, and parameters are changed in the pooling kernel [5].

Let the target image be *F*(*m*, *n*), *m* is the width of the image, and *n* is the height of the image. The pooling domain is *f*(*i*, *j*), where *i* = 1, 2, ⋯, *n* and *j* = 1, 2, ⋯, *m*, which is the size of the receptive field in the pooling process. The pooling kernel is *w*(*i*, *j*), where *i* = 1, 2, ⋯, *n* and *j* = 1, 2, ⋯, *m*, which is the filter of the pooling domain. The pooling step length is the step length of the pooling nucleus every time it moves. The activation value *x*_*i*,*j*_ is the feature value of the *i*-th row and *j*-th column in the feature map. The pooling feature value of the *i*-th row and *j*-th column is obtained by pooling operation.

In the pooling domain of size *i* × *j*, the value in the pooling domain is determined by the feature map. The pooling domain *f*(*i*, *j*) is determined by the pooling kernel *w*(*i*, *j*), and the pooling kernel is a matrix of size *i* × *j*. Generally, the pooling kernel has no parameters. The pooling domain *f*(*i*, *j*) can be represented as

(1) $$\begin{array}{c} f\left(i,j\right)={\left[\begin{array}{cccc} x_{p,q} & x_{p,q+1} & \cdots & x_{p,q+j}\\ x_{p+1,q} & x_{p+1,q+1} & \cdots & x_{p+1,q+j}\\ \vdots & \vdots & \ddots & \vdots \\ x_{p+i,q} & x_{p+i,q+1} & \cdots & x_{p+i,q+j} \end{array}\right]}_{i\times j}. \end{array}$$

It can be concluded that the response feature map *g*(*i*, *j*) is determined by the pooling kernel *w*(*i*, *j*) in the feature map *F* of size *m* × *n*. It is expressed as

(2) $$\begin{array}{c} g\left(i,j\right)={\left[\begin{array}{cccc} y_{1,1} & y_{1,2} & \cdots & y_{1,j}\\ y_{2,1} & y_{2,2} & \cdots & y_{2,j}\\ \vdots & \vdots & \ddots & \vdots \\ y_{i,1} & y_{i,2} & \cdots & y_{i,j} \end{array}\right]}_{i\times j},\\ y_{i,j}=\beta \text{down}\left(x\left(i,j\right)\right)+b, \end{array}$$

where down(.) represents the downsampling operation, which is the pooling kernel *w*(*i*, *j*) for the pooling operation of values in the pooling domain *f*(*i*, *j*). *β* is the pooling coefficient. *y*_*i*,*j*_ is the response value of the pooling domain. The response value is the value of the response feature map in the *i*-th row and *j*-th column. And *b* is the bias value of pooling operation.

**Figure 2:**
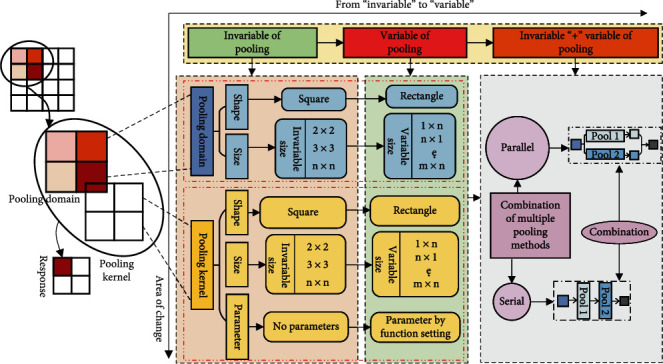
“Invariable” to “variable” of pooling operation.

The development of pooling operation is a process from “invariable” to “variable,” as shown in Figure 2. The most commonly used pooling operation is max pooling operation and average pooling operation. Max pooling operation and average pooling operation are calculated simply. They have few parameters and work well on most tasks. So far, these pooling operations are still the most commonly used pooling methods. In addition, max pooling operation and average pooling operation cannot achieve good results on specific tasks. Many scholars have further studied the pooling operation for different problems. Firstly, change the value of pooling selection. Secondly, change the pooling domain, which is including the change of shape and size. Thirdly, change the pooling kernel, which is including the change in shape, size, and parameters. Finally, there are various pooling methods that are combined, which have parallel methods and serial methods. The parallel method is the most commonly used.

**Figure 3:**
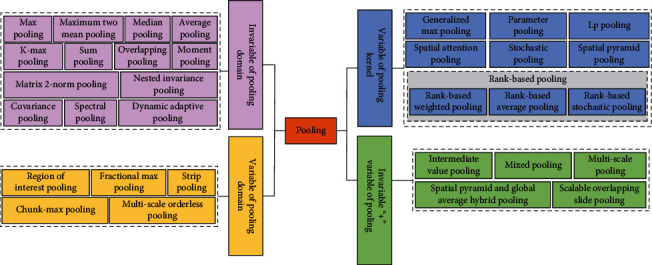
Summary of pooling methods.

In this paper, the “variable” and “invariable” of the pooling operation are divided into four categories: invariable of pooling domain, variable of pooling domain, variable of pooling kernel, and the invariable “+” variable of pooling. The overall framework is shown in Figure 3. The invariable of pooling domain means that there is no change in the shape and size of the pooling domain. The main methods are max pooling operation, maximum two mean pooling operation, *K*-max pooling operation, average pooling operation, median pooling operation, sum pooling operation, overlapping pooling operation, matrix 2-norm pooling operation, moment pooling operation, covariance pooling operation, dynamic adaptive pooling operation, and nested invariance pooling operation. The variable of pooling domain means that there is change in the shape and size of the pooling domain. The main methods are region of interest pooling method, fractional max pooling method, strip pooling method, chunk-max pooling method, and multiscale orderless pooling method. The variable of pooling kernel means that there is change in the parameters, selection method, and size of the pooling kernel. The main methods are generalized max pooling operation, parameter pooling operation, LP pooling operation, spatial attention pooling operation, stochastic pooling operation, spatial pyramid pooling operation, and rank-based pooling (rank-based average pooling operation, rank-based weighted pooling operation, and rank-based stochastic pooling operation) operation. The invariable “+” variable of pooling means that there are two or more pooling methods in pooling methods, including variable pooling operation and invariable pooling operation. The main methods are intermediate value pooling operation, mixed pooling operation, spatial pyramid and global average hybrid pooling operation, multiscale pooling operation, and scalable overlapping slide pooling operation.

## 3. Invariable of Pooling Domain

**Figure 4:**
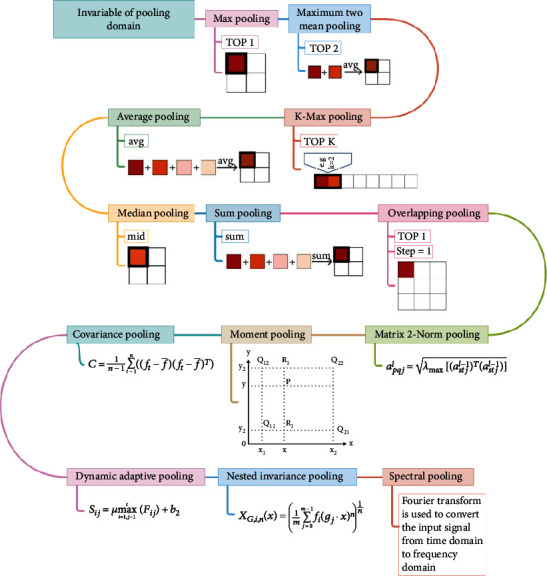
Invariable of pooling domain.

Invariable of pooling domain means that there is no change in the shape and size of the pooling domain. And there is change in the shape, size, and parameters of the pooling kernel. In this paper, the variable of pooling domain is divided into two methods: basic pooling operation and improved pooling operation. The basic pooling operation has the advantages of simple calculation process and wide application range. But the utilization rate is low in the feature map. The improved pooling operation uses different mapping rules to select the eigenvalues in the pooling domain. So the improved pooling operation can improve the utilization rate of the feature maps. But the calculation process is more complicated. The development process of invariable of pooling domain is shown in Figure 4.

### 3.1. Basic Pooling Operation

The basic pooling operation during the pooling operation is that the shape and size of the pooling domain are unchanged, and the shape, size, and parameters of the pooling kernel are unchanged. Select the eigenvalues with different numbers in the pooling domain, including max pooling operation, maximum two-mean pooling operation, *K*-max pooling operation, average pooling operation, median pooling operation, sum pooling operation, and overlapping pooling operation.

Firstly, max pooling operation selects the largest activation value in the pooling domain as the output response value. Other activation values are discarded at the same time. The advantage of max pooling operation is that salient features can be extracted, and the contour information of the image can be learned. However, max pooling operation loses too much features and information. The global max pooling operation is a case that max pooling operation is generalized to the global. The difference is that the size of the pooling domain is the same as the size of the entire feature map. Secondly, maximum two-mean pooling operation selects the two largest activation values in the pooling domain and averages them. Use the averaged value as the response value. The maximum two-mean pooling operation improves the disadvantage of ignoring features with larger influencing factors of max pooling. But there is some information missing. Thirdly, *K*-max pooling operation extracts the top *K* values in the pooling domain as the response value and preserves the order of these values. *K*-max pooling operation retains more information than max pooling, which can express the strength of a certain type of feature. *K*-max pooling operation retains position information, but it is the relative order between features, not absolute position information. Fourthly, average pooling operation means that the average of activation values in the pooling domain and the average value are used as the response value. Average pooling considers the global information, and there are no information loss and reducing overfitting. But it makes the features smooth and cannot extract the information of prominent features. The global average pooling operation is a case that average pooling operation is generalized to the global. The difference is that the size of the global average pooling domain is the same as the size of the entire feature map. Fifthly, median pooling operation takes the median of all activation values in the pooling domain as the response value. Median pooling operation has the characteristics of learning edge and texture structures and has a strong ability to resist noise, which can improve performance. Sixthly, sum pooling operation takes the sum value of all activation values in the pooling domain as the response value. Sum pooling operation can be applied to any encoding, and the impact of frequent descriptors is greater than that of rare descriptors. Seventhly, overlapping pooling [6] operation selects the largest activation value in the pooling domain as the output response value. The pooling domain of overlapping pooling operation has overlapping parts because its stride is 1. The overlapping pooling operation augments the output into multiple levels of smaller features. Overlapping pooling operation uses sparse coding to fuse multilevel features, which reduces the feature dimension of the output and improves the network performance.  Table 1 shows the summary of basic pooling operation.

### 3.2. Improved Pooling Operation

The improved pooling operation means that there are no change in the shape and size of the pooling domain and change in the shape, size, and parameters of the pooling kernel. But the difference is the way to get the response value of eigenvalues in the pooling domain using the mapping rule. Improved pooling operation includes matrix 2-norm pooling operation, moment pooling operation, covariance pooling operation, dynamic adaptive pooling operation, nested invariance pooling operation, and spectral pooling operation.

Firstly, matrix2-norm pooling [7] operation makes the eigenvalues of feature map as the coefficient matrix of the singular equation system. Take the square root of the largest singular value obtained from this matrix as the response value of the pooling domain. When the image is geometrically distorted such as rotation, translation, and scaling, there are little changes in the singular value of the image matrix. This means that the energy information of the image has high stability, so the singular value of the matrix can make the geometric distortion of the image highly invariant. Secondly, moment pooling [8] operation calculates the central moment *c*(*i*, *j*) of the pooling domain using the gray moment. Calculates the four neighborhoods of the central moment *c*. Calculate the probability of each neighborhood using the distances between the four neighborhoods *x*(*i*, *j*), *x*(*i* + 1, *j*), *x*(*i*, *j* + 1), *x*(*i* + 1, *j* + 1), and center *c*(*i*, *j*). The closer the distance is, the greater the probability of being selected, which is conducive to the probability of selecting the response value. Because the randomness of moment pooling operation makes each selection different, it can effectively prevent oversuppression. Moment pooling operation randomly generates 2 probabilities. The computational complexity of the moment pooling operation is much lower than bilinear interpolation and close to the nearest neighbor interpolation. Thirdly, covariance pooling [9] operation calculates the covariance matrix using the eigenvalue matrix of the feature map and then adjusts the matrix to what you need. For example, the matrix is normalized to get the response values. Covariance pooling operation [10] belongs to the second-order pooling method, which can capture more information about the feature map than most first-order pooling methods. Fourthly, dynamic adaptive pooling operation [11] optimizes max pooling operation with a pooling factor *μ*. Dynamic adaptive pooling operation builds a mathematical model for function simulation, and other parameters are the same as max pooling operation. The pooling factor will not lose accuracy when it deals with pooling domains with obvious max characteristics. It can weaken the influence of max pooling operation when it deals with other pooling domains. Therefore, the CNN can extract more accurate features under different iterations. Fifthly, nested invariance pooling [12] operation changes the output of feature maps in the pooling domain using invariant transformation, which is the response feature map. It can be extended to any transform set. Lou et al. [13] obtain global descriptors by concatenating all feature maps. They construct nested invariance pooling operation from existing local translation invariant feature maps. Zhang et al. [14] showed that various types of transformation invariants can be linked. Sixthly, spectral pooling [15, 16] operation is performed for dimensionality reduction by projecting onto the frequency basis set and then truncating the representation. It is a pooling method based on fast Fourier transform, which keeps the same number of parameters and retains more information. Spectral pooling operation can be regarded as a denoising method that allows specifying any arbitrary output feature dimension. The truncation of frequency representation corresponds to the reduction in resolution. So spectral pooling operation can be supplemented with random regularization in the form of random resolution.  Table 2 shows the summary of improved pooling operation.

## 4. Variable of Pooling Domain

**Figure 5:**
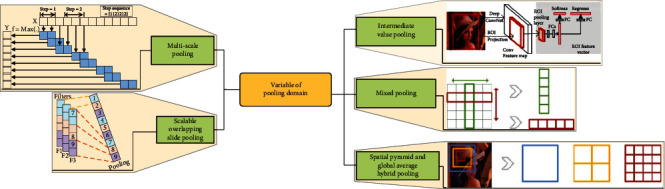
Variable of pooling domain.

Variable of pooling domain means that there is change in the shape and size of the pooling kernel. And there is no change in the parameters of the pooling kernel. The shape and size of the pooling kernel are determined by the pooling domain. Variable of pooling domain can modify the size and shape of the pooling domain as needed, which is more flexible. But it leads to susceptibility to more irrelevant pixels when the pooling domain changes. Variable of pooling domain includes region of interest pooling operation, fractional max pooling operation, strip pooling operation, chunk-max pooling operation, and multiscale orderless pooling operation, as shown in Figure 5.

**Figure 6:**
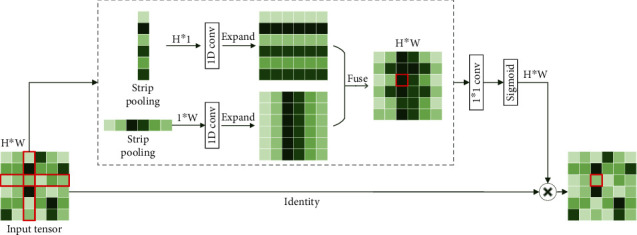
Schematic diagram of strip pooling [21].

Firstly, region of interest pooling (ROI pooling) [17] is pooling just for the region of interest. There is no fix in the shape and size of the pooling domain which is depending on the region of interest. The pooling domain is the region of interest in which a max pooling operation in the pooling domain is done. ROI pooling operation was used in fast RCNN [17, 18] first. Its advantage is that the location area and shape size of the pooling domain can be determined due to the problem, and the size of the output feature map can be determined. And just the region of interest in the feature map needs to be pooled, which greatly reduces the amount of computation. In the calculation process, the position of the region of interest is easily selected due to the quantization operation. Secondly, fractional max pooling [19] operation divides the feature map of input size *b* × *b* into *a* × *a* blocks. These feature blocks of the *a* × *a* are *a* × *a* pooling domains, and then, select the max value of each pooling domain to obtain the response value. Fractional max pooling operation allows the pooling domain to be noninteger values. It avoids the problem of limited performance due to disjoint pooling kernels when the pooling domain is integer. Yue et al. [20] make random number sequences or pseudorandom sequences as the size of each pooling domain, and the integer sequence generates 1 or 2. It reduces the overfitting of the dataset due to the randomness of the sequence. Thirdly, strip pooling [21] operation performs pooling operations along the horizontal or vertical dimension using a strip pooling domain. The pooling domain of strip pooling operation is an *h* × 1 or 1 × *w* strip domain, and average values in the pooling domain rowwise or columnwise are shown in Figure 6. Strip pooling alleviates the problem of merging irrelevant regions in the process of processing objects with irregular shapes about average pooling. It can be easily embedded into any building block without training from scratch because the strip pooling module is lightweight. Strip pooling has long and narrow pooling field shapes. It is easy to establish long-range dependencies between discretely distributed regions and encode the strip-shaped regions. Fourthly, chunk-max pooling [22] operation segments the feature vector on the feature map as pooling domain. Chunk-max pooling operation performs max pooling operation for each pooling domain, obtaining multiple response values. This method of segmenting eigenvectors can be set in advance or dynamically divided according to eigenvalues. Static segmentation means that the number of paragraphs can be set in advance. Dynamic segmentation means that the boundary positions between chunks can be dynamically divided according to different inputs. Chunk-max pooling operation retains relatively coarse-grained fuzzy location information. Feature intensity can be captured if a strong feature occurs multiple times. Fifthly, multiscale orderless pooling [23] operation extracts deep activation features through three pooling domains of different sizes. Then, the local responses are aggregated on a finer scale by local aggregation vector (VLAD) coding. Finally, the original global deep activations are connected with the VLAD feature to form response value. The features obtained after activation of global CNNs lack geometric invariance, which includes rotation invariance and translation invariance. Multiscale orderless pooling operation is proposed to solve this problem. It limits their robustness to the classification and matching of variable scenes.  Table 3 shows the summary of variable of pooling domain.

## 5. Variable of Pooling Kernel

**Figure 7:**
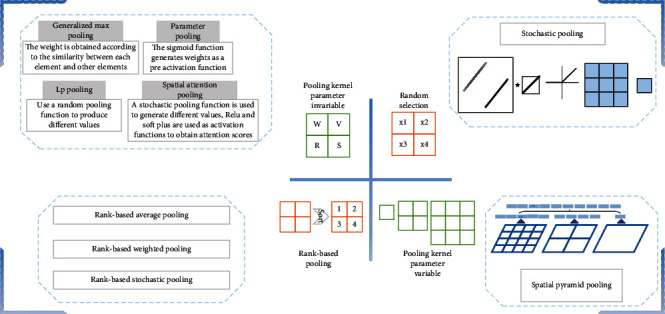
Variable of pooling kernel.

Variable of pooling kernel means that there is change in the shape, size, and parameters of the pooling kernel. Variable of pooling kernel can get a better solution for the problem to be solved, and the pooling changes the pooling kernel. In this paper, the variable of pooling kernel is divided into four categories: pooling kernel parameter invariable, random selection, rank-based pooling, and pooling kernel parameter variable, as shown in Figure 7. The pooling kernel parameter invariable is to set the parameters of the pooling kernel. The response value is obtained by multiplying the parameters in the pooling kernel with the activation value in the pooling domain. Random selection randomly selects values in the pooling domain as the output response value. Rank-based pooling sorts all the values in the pooling domain and then performs averages, weights, or random pooling on the ordered activation values. The pooling kernel parameter variable is the improvement of the pooling operation in front of the fully connected layer. The pooling kernel parameter variable uses three pooling kernels of different sizes to generate a fixed-length feature vector for the max pooling operation. The obtained result is passed to the fully connected layer.

**Figure 8:**
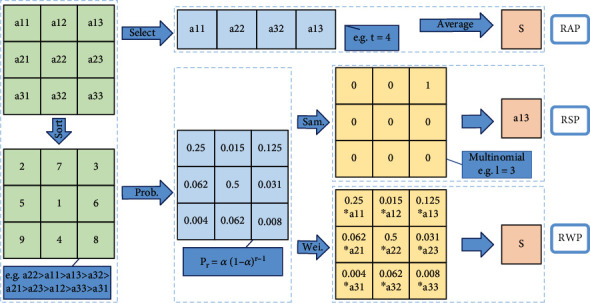
Rank-based pooling [28].

Firstly, generalized max pooling [24] operation is a process of obtaining weights by solving the optimization problem. The weights are obtained according to the similarity between each element and other elements. Rare pixels with lower similarity will have larger weights. Finally, each element will be weighted and summed as the output. Generalized max pooling operation achieves the same effect as max pooling operation. It is also suitable for places other than the visual word package (BOV), especially for the Fisher vector. Secondly, parameter pooling [25] operation uses the operation rules of the pooling kernel parameters to obtain the parameters of the pooling kernel. The parameters of the pooling kernel are matrix multiplied by the activation value to obtain the response value. Generally, the pooling kernel has no parameters. Parameter pooling operation allows the model to choose the pooling method autonomously because it has a few parameters. Parameter pooling operation preserves feature information as much as possible. It reduces network errors, and the network performance is improved. Thirdly, LP pooling [26] operation uses a Gaussian kernel to weight the pixel values of the input feature map. At this time, the Gaussian kernel is the pooling kernel of the pooling operation, and the weighted value is the response value. The optimal pooling method for most problems is neither average pooling operation nor max pooling operation but a certain type between them. When *p* = 2, it is L2 pooling operation. There are good results that can be achieved in most image classification problems. Fourthly, spatial attention pooling [27] operation generates an attention parameter matrix for the entire feature map. The parameter matrix acts as a pooling kernel to perform a global pooling operation on the feature map. The value on the attention parameter matrix is multiplied by the values of the feature map to obtain a new matrix as the response result of spatial attention pooling operation. Spatial attention pooling operation is able to highlight regions that require attention. Fifthly, rank-based pooling [28] operation sorts the eigenvalues in the pooling domain from large to small. It performs average pooling operation, weighted pooling operation, and stochastic pooling operation on the sorted values, as shown in Figure 8. The traditional pooling methods operate directly on the values in the feature map. There are many possible values in a pooling domain, but the ranking of eigenvalues is relatively stable. Therefore, rank-based pooling operation can avoid the influence caused by the change of the value. Rank-based average pooling [29] (RAP) operation sorts the values of the feature maps from large to small and selects the *t* with the largest value to average the response value. RAP can alleviate the problem of information loss from max pooling operation and discriminative information loss from average pooling operation. Rank-based weighted pooling [30] (RWP) operation assigns weight *p*_*r*_ according to the size of the value. The value as the response value is obtained by multiplying the feature map matrix and the weight matrix. It can be assigned the larger weights for the higher activation value. The value ordering method of RWP can be a linear function or a nonlinear function. RWP assigns a reasonable weight value to each activation value in the pooling domain, which can improve the performance significantly. Rank-based stochastic pooling [31] (RSP) computes the probability *p*_*r*_ using exponential ranking on the resulting ranking values. It selects activation values by sampling from a multinomial distribution based on *p*_*r*_. It is proposed to solve the problem that random pooling is just suitable for nonnegative values, and feature probability calculated by standardized eigenvalues is out of control, which is easy to lead to overfitting.

Sixthly, stochastic pooling [29] operation obtains the probability according to the value. The probability is used to select randomly, and the selected activation value is output. The higher the probability value, the greater the probability of being selected. Stochastic pooling operation is the process of random selection. When the selected pooling value is the max value in the pooling domain, the calculation process is equivalent to max pooling operation. Seventh, spatial pyramid pooling [33] operation performs max pooling operations on feature maps at three different scales. It replaces the last pooling layer with a spatial pyramid pooling layer, which can process information of different scales in the image to generate the fixed length feature information. Spatial pyramid pooling avoids the fact that most CNNs need to input fixed size images artificially, thus solving the limitation of image recognition accuracy due to fixed size. No matter how the feature map size changes, as long as the output length is fixed, the final number of features will be fixed. Images of different scales can be processed by Spatial Pyramid Pooling operation, so it is very flexible and can effectively prevent overfitting. However, some problems cannot be calculated in spatial pyramid pooling operation. When setting the output length, it is necessary to take into account the limitations of the actual situation.  Table 4 shows the summary of variable of the pooling kernel.

## 6. The Invariable “+” Variable of Pooling

**Figure 9:**
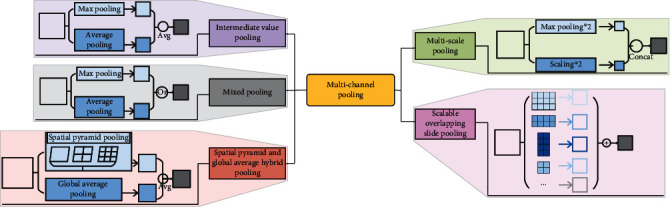
The pooling of invariable “+” variable.

The invariable “+” variable of pooling means that it is a pooling method that uses different pooling methods for pooling separately and then fuses multiple pooling results. It can also be called multichannel pooling. Multichannel pooling operation means that there are two or more pooling methods in pooling methods to compensate for the defects of a single pooling operation. However, at the same time, it will increase the amount of computation. Multichannel pooling includes intermediate value pooling operation, mixed pooling operation, spatial pyramid and global average hybrid pooling operation, multiscale pooling operation, and scalable overlapping slide pooling operation, as shown in Figure 9.

**Figure 10:**
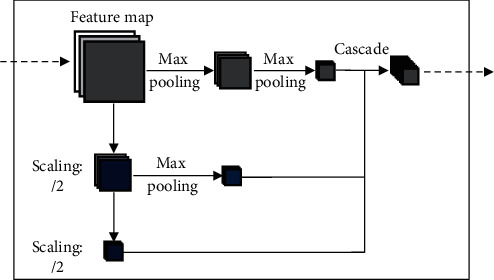
Multiscale pooling.

Firstly, intermediate value pooling [34] operation is used to perform max pooling operation and average pooling operation on the same feature map in parallel. The response values of the two pooling methods are averaged to obtain the response feature map. It combines the characteristics of both and highlights the most discriminative features while preserving the information integrity of the feature map. It is generally suitable for general images, because the error of the model is smaller and the stability is higher. Secondly, mixed pooling [35] operation is random using max pooling operation or average pooling operation. Mixed pooling operation utilizes random sampling instead of deterministic pooling operations. To some extent, mixed pooling operation solves the problem of which to choose between max pooling operation and average pooling operation. It can greatly improve the overall performance of the model when combined with other forms of regularization methods. Thirdly, spatial pyramid and global average hybrid pooling [36] operation uses spatial pyramid pooling operation and global average pooling operation for pooling feature maps in parallel. There are two pooling methods fused to get response values. Before fully connecting, global average pooling operation can reduce parameters, dimensionality, and overfitting without destroying the spatial characteristics of feature maps. Spatial pyramid pooling operation shows the information of local characteristics. Global average pooling shows the information of global characteristics. There are two pooling methods fused that can learn more comprehensive information. Fourthly, multiscale pooling operation performs max pooling operation twice serially. Then, perform scale operation twice serially. Next, perform max pooling operation and scale operation serially. Finally, concatenate the feature maps obtained by three pooling methods as the response value, as shown in Figure 10. Multiscale pooling operation replaces the max pooling operation in ResNet, which is used to improve the recognition effect of images collected at different resolutions. Compared with the spatial pyramid model, the advantage of multiscale pooling operation is that its substitution position is more flexible and it can be used many times at the beginning, middle, or end of a network. Fifthly, scalable overlapping slide pooling [37] operation pools the feature maps in parallel through pooling kernels of multiple scales. The overlapping slide pooling operation obtains coarse-grained, medium-grained, and fine-grained feature maps. Finally, the multiscale pooling operation response feature maps of multiscale pooling are spliced as the response value. Single-scale pooling operation does not consider the saliency of features in different scales and the relationship between adjacent feature elements, which leads to decreased inaccuracy. Multiscale pooling operation solved this problem. More comprehensive and accurate granular features can be obtained through multiscale overlapping sliding pooling operation. The detection ability of the network for small targets and the overall detection accuracy can be improved.  Table 5 shows the summary of multichannel pooling.

## 7. Conclusions

With the continuous development of multimedia technology, deep learning is widely used in text, speech, image, video, etc. Among them, pooling operation in deep learning has been deeply studied. And pooling operation is iterated from “invariable” to “variable,” with different problems and tasks. These methods are constantly optimized due to different problems and tasks. Now, the most common methods of pooling are max pooling operation and average pooling operation. The newly proposed pooling method can improve the efficiency, improve the accuracy, and reduce the degree of overfitting.

In this paper, pooling operation in deep learning is comprehensively summarized. And introduce the pooling steps and their characteristics in detail and standardize the expression of pooling operation. The pooling operation is divided into the pooling domain, pooling kernel, step size, activation value, and response values. The improvement of pooling domain and pooling kernel in pooling operation is summarized as “variable” and “invariable” of pooling operation. Pooling operation can be classified into four categories: invariable of pooling domain, variable of pooling domain, variable of pooling kernel, and the pooling of invariable “+” variable. Invariable of pooling domain refers to the shape of the pooling domain being invariable in pooling operation. There are two types of invariable in pooling domain, including basic pooling methods and improved pooling methods. Variable of pooling domain means that shape and size of pooling domain are changed. Variable of pooling kernel means that the parameters, selection methods, and size of pooling kernel are changed. Pooling of invariable “+” variable means that there are two or more pooling methods in pooling methods. Although pooling operation is constantly being researched, deep learning is developing increasingly better, which has a better role for further research in deep learning.

## Figures and Tables

**Table 1:** Summary of basic pooling operation.

Pooling method	Characteristic	Sketch map
Max pooling	The max pooling operation is simple, but the features with strong influencing factors are ignored and lost information	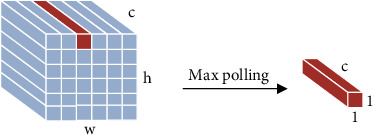
Maximum two-mean pooling	Maximum two-mean pooling operation improves the disadvantage of max pooling to a certain extent, which is ignoring features with larger influencing factors. But there is some problem of loss information	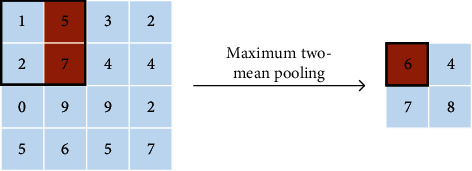
*K*-max pooling	*K*-max pooling operation preserves more information than max pooling and preserves the relative order of eigenvalues, but there is no absolute position information	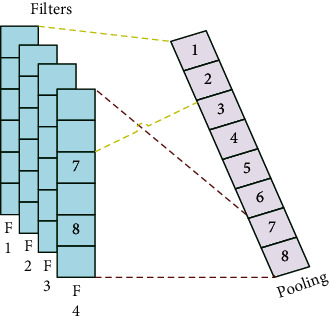
Average pooling	Average pooling operation takes global information into account, no information is lost, and overfitting is reduced. However, the features tend to be smooth, and the information of prominent features cannot be extracted	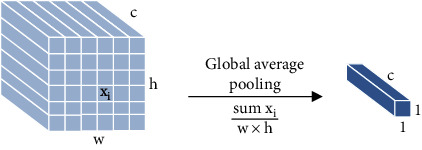
Median pooling	Median pooling operation can learn the characteristics of edge and texture structure and has a strong antinoise ability	*Y*(*F*) = Median(*S*_1_, *S*_2_, ⋯, *S*_*N*_) *S*_*i*_ = Median(*f*_*i*1_, *f*_*i*1_, ⋯, *f*_*im*_*i*__) *i* = 1, 2, ⋯*N*
Sum pooling	Sum pooling operation considers global information. There is no information loss, but it is vulnerable to extreme information	—
Overlapping pooling [6]	Overlapping pooling operation reduces the characteristic dimension of the output and reduces overfitting, but there are information that will be lost	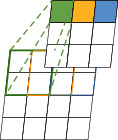

**Table 2:** Summary of improved pooling operation.

Pooling method	Characteristic	Sketch map
Matrix2-norm pooling [7]	Matrix 2-norm pooling operation takes the energy of the image as the information transmitted to the next layer network to make geometric distortion of the image is highly invariable	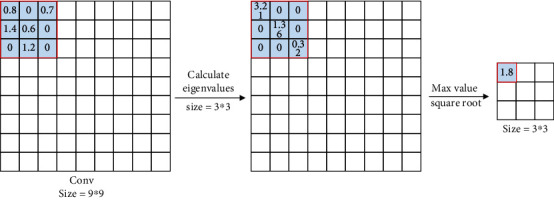
Moment pooling [8]	The randomness of moment pooling operation makes each choice different, to prevent over inhibition	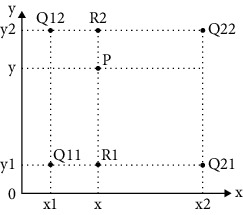
Covariance pooling [9]	Covariance pooling operation can capture more information on the feature map	$C=\left(1/n-1\right)\sum_{i=1}^{n}\left(\left(f_{i}-\overset{\_}{f}\right){\left(f_{i}-\overset{\_}{f}\right)}^{T}\right)$
Dynamic adaptive pooling [11]	Dynamic adaptive pooling operation can adaptively adjust the weight of pooling and extract more accurate features	$y_{ij}=\mu \overset{c}{\underset{i=1,j=1}{\max}}\left(x_{ij}\right)+b_{2}$ *μ* = *ρ*(*λ*(*x*_max_ − *λ*)/*x*_max_^2^) + *θρ* = *c*/1 + (*n*_*epo*_ − 1)*c*^*n*_*epo*_^2^+1^
Nested invariance pooling [12]	Nested invariance pooling operation can be extended to any arbitrary transformation set, allowing any arbitrary output feature dimension to be specified	*Y*_*G*,*i*,*n*_(*x*) = ((1/*m*)∑_*j*=0_^*m*−1^*f*_*i*_(*g*_*j*_.*x*)^*n*^)^1/*n*^
Spectral pooling [15]	Spectral pooling operation has higher efficiency and lower cost	—

**Table 3:** Summary of variable of pooling domain.

Pooling method	Characteristic	Sketch map
Region of interest pooling [17]	ROI pooling operation converts feature maps within a region of interest of any size into feature maps of fixed size	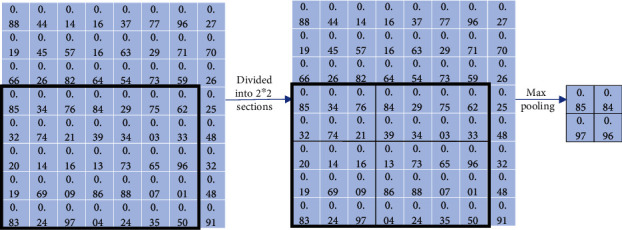
Fractional max pooling [19]	Fractional max pooling operation allows the pooling domain to be noninteger values and reduces overfitting	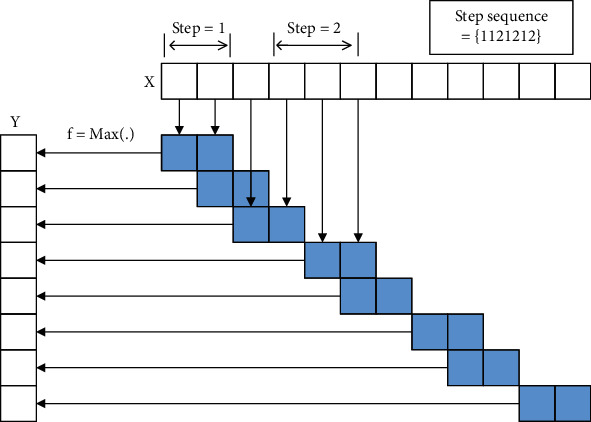
Strip pooling [21]	Strip pooling operation is easy to establish remote dependencies between discrete distributed regions. It can capture local details and can be easily embedded into any building block	*y*_*i*_^*h*^ = 1/*w*∑_0≤*j*≤*W*_*x*_*i*,*j*_, *y*_*j*_^*v*^ = 1/*H*∑_0≤*i*≤*H*_*x*_*i*,*j*_
Chunk-max pooling [22]	Chunk-max pooling operation retains the relative order information of multiple local max eigenvalues but does not retain the absolute position information	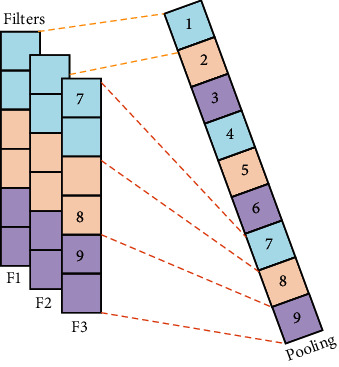
Multiscale orderless pooling [23]	Multiscale orderless pooling operation improves the invariance of neural network activations without reducing its resolution	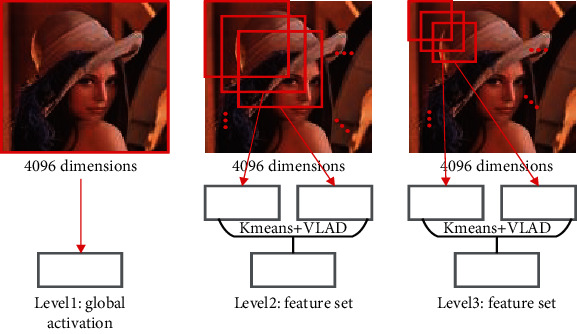

**Table 4:** Summary of variable of the pooling kernel.

Pooling method	Characteristic		Sketch map
Generalized max pooling [24]	Generalized max pooling operation balances the influence of frequent pixels and rare data and improves the ability to extract fine-grained data		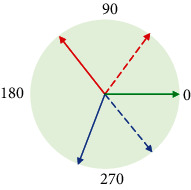
Parameter pooling [25]	Parameter pooling operation converts the correlation operation into an interpretable pooling operation, which retains information and reduces errors		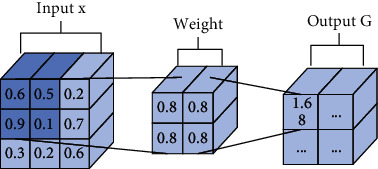 *Y*_*c*_(*i*, *j*) = ∑_*m*=0_^*p*^∑_*n*=0_^*q*^*F*_*c*_(*i* + *m*, *j* + *n*) · *β*(*W*_*c*_(*m*, *n*))
LP pooling [26]	LP pooling operation balances the effects of max pooling and average pooling.		*Y* = (∑∑*F*(*i*, *j*)^*P*^ × *G*(*i*, *j*))^1/*P*^
Spatial attention pooling [27]	Spatial attention pooling operation mitigates the effects of distracting factors and focuses on meaningful parts of the image		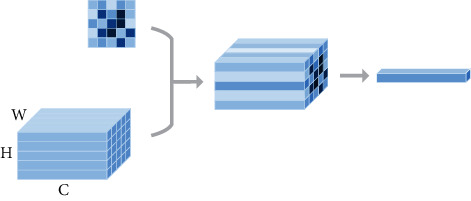
Rank-based pooling [28]	Rank-based average pooling	Alleviate the problem of loss of information in max pooling and loss of discriminative information in average pooling	*y*_*j*_ = (1/*t*)∑_*i*∈*f*_*j*_,*r*_*i*_≤*t*_*x*_*i*_
	Rank-based weighted pooling	Assigning a weight value to each pixel in the pooling domain can improve performance	*p*_*r*_ = *α*(1 − *α*)^*r*−1^ *y*_*j*_ = ∑_*i*∈*f*_*j*__*p*_*i*_*x*_*i*_
	Rank-based stochastic pooling	Alleviates the problem that random pooling is limited to nonnegative values and reduces overfitting	*p*_*r*_ = *α*(1 − *α*)^*r*−1^ *y*_*j*_ = *x*_*l*_
Stochastic pooling [32]	Stochastic pooling operation is simple and has a strong generalization ability		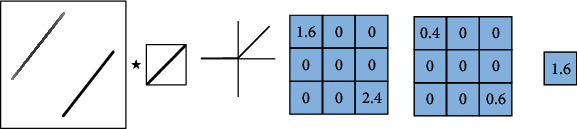 *p*_*i*_ = *x*_*i*_/∑_*k*∈*f*_*j*__*x*_*k*_ *y*_*j*_ = *x*_*l*_, where *l* ~ *P*(*p*_1_, ⋯, *p*_|*R*_*j*_|_).
Spatial pyramid pooling [33]	Spatial pyramid pooling operation can handle images of different scales, is very flexible to use, and can effectively prevent overfitting. However, there are also practical constraints to consider		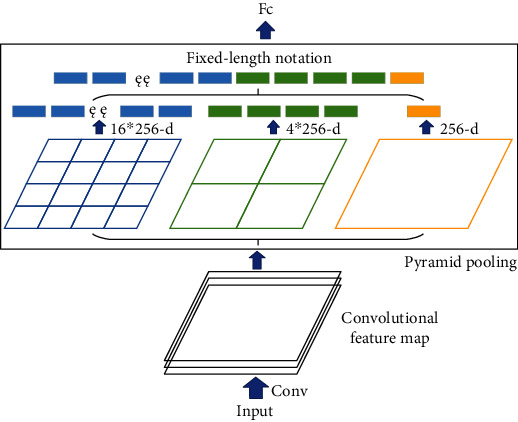

**Table 5:** Summary of multichannel pooling.

Pooling method	Characteristic	Sketch map
Intermediate value pooling [34]	It takes into account the average pooling and the max pooling, so that it has smaller model error and higher stability	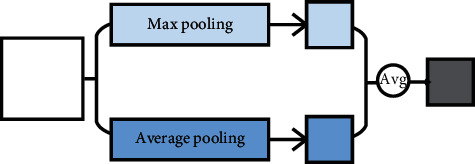 *Y* = 1/2 × (AvgPool(*F*) + MaxPool(*F*))
Mixed pooling [35]	It solves the problem of which to choose between max pooling and average pooling, but only using one of these pooling methods still has the disadvantage of max pooling or average pooling	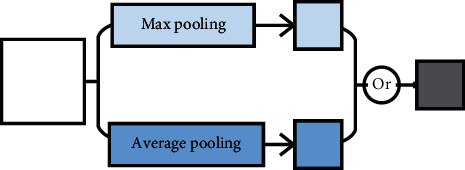 *y*_*kij*_ = *λ*max(*x*_*kqp*_) + (1 − *λ*)(1/|*f*_*ij*_|) · ∑*x*_*kqp*_ (*p*, *q*) ∈ *f*_*ij*_
Spatial pyramid and global average hybrid pooling [36]	It can extract local and global information, respectively, which learns more information	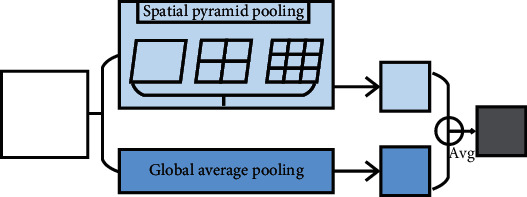
Multiscale pooling	It is more flexible and can be used multiple times at the beginning, middle, or end of the network	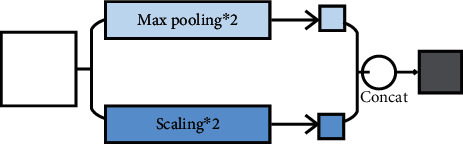
Scalable overlapping slide pooling	It considers the saliency of features at different scales and the relationship between adjacent feature elements, so that coarse-grained, medium-grained, and fine-grained multiple features can be extracted	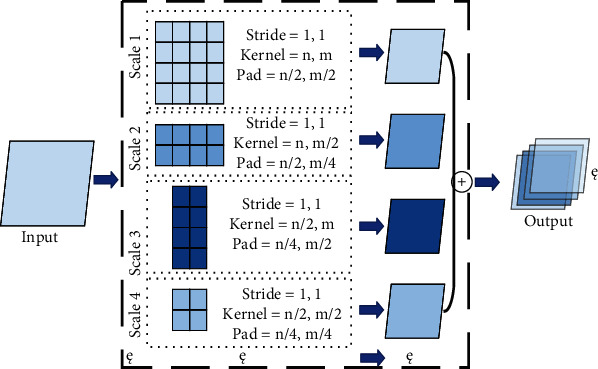

## Data Availability

The data that support the findings of this study are available from the corresponding author.

## References

[1] Hinton G. E., Salakhutdinov R. R. (2006). Reducing the dimensionality of data with neural networks. *Science*.

[2] Le C., Boser Y., Denker B. (1989). Backpropagation applied to handwritten zip code recognition. *Neural Computing*.

[3] Zhou T., Ye X., Lu H., Zheng X., Qiu S., Liu Y. (2022). Dense convolutional network and its application in medical image analysis. *BioMed Research International*.

[4] Zhou T., Lu H., Yang Z., Qiu S., Huo B., Dong Y. (2021). The ensemble deep learning model for novel COVID-19 on CT images. *Applied Soft Computing*.

[5] Tao Z., Bing-Qiang H., Huiling L., Hongbin S., Pengfei Y., Hongsheng D. (2021). 18F-FDG PETCT whole body imaging lung tumor diagnostic model: an ensemble E-Res Net-NRC with divided sample space. *BioMed Research International*.

[6] Krizhevsky A., Sutskever I., Hinton G. E. (2017). ImageNet classification with deep convolutional neural networks. *Communications of the ACM*.

[7] Li T., Meng Z., Ni B., Shen J., Wang M. (2016). Robust geometric ℓp-norm feature pooling for image classification and action recognition. *Image and Vision Computing*.

[8] Otsuzuki T., Hayashi H., Zheng Y., Uchida S. (2020). Regularized pooling. *Artificial Neural Networks and Machine Learning-ICANN 2020*.

[9] Li P., Xie J., Wang Q., Gao Z. Towards faster training of global covariance pooling networks by iterative matrix square root normalization.

[10] Acharya D., Huang Z., Paudel D. P., Van Gool L. Covariance pooling for facial expression recognition.

[11] Wanjun L., Liang X., Haicheng Q. (2016). Learning performance of CNNs with different pooling models. *Journal of Image and Graphics*.

[12] Morère O., Lin J., Veillard A., Duan L. Y., Chandrasekhar V., Poggio T. Nested invariance pooling and RBM hashing for image instance retrieval.

[13] Lou Y., Bai Y., Lin J. Compact deep invariable descriptors for video retrieval.

[14] Zhang C., Evangelopoulos G., Voinea S. C., Rosasco L., Poggio T. A deep representation for invariance and music classification.

[15] Rippel O., Snoek J., Adams R. P. (2015). Spectral representations for CNNs, International Conference on Neural Information Processing Systems. https://arxiv.org/abs/1506.03767.

[16] Hao Z., Ma J. (2020). Hartley spectral pooling for deep learning. *CSIAM Transactions on Applied Mathematics*.

[17] Sun Y., Sun C., Wang D., He Y., Lu H. ROI pooled correlation filters for visual tracking.

[18] Zhu Y., Xu T., Peng L. (2022). Faster-RCNN based intelligent detection and localization of dental caries. *Displays*.

[19] Raymond F. E., Raymond B. W. (2015). Voluntarily relinquishing private property rights: the existence of risk-pooling wquilibria when facing environmental uncertainty. *Theoretical Economics Letters*.

[20] Kaiyu Y., Xu F., Yu J. (2019). Shallow and wide fractional max-pooling network for image classification. *Neural Computing and Applications*.

[21] Hou Q., Zhang L., Cheng M. M., Feng J. Strip pooling: rethinking spatial pooling for scene parsing.

[22] Lin J., Ma L., Jingxia C. (2020). A frequency-domain CNN architecture based on the frequency-domain randomized offset rectified linear init and frequency-domain chunk max pooling method. *IEEE Access*.

[23] Gong Y., Wang L., Guo R., Lazebnik S. Multi-scale orderless pooling of deep convolutional activation features.

[24] Christlein V., Spranger L., Seuret M., Nicolaou A., Král P., Maier A. Deep generalized max pooling.

[25] Zetao J., Jiaqi Q., Shaoqin Z. (2020). Parameterized pooling convolution neural network for image classification. *Acta Electronica Sinica*.

[26] Sermanet P., Chintala S., Lecun Y. CNNs applied to house numbers digit classification.

[27] Ma J., Xiaodong G. (2020). Scene image retrieval with siamese spatial attention pooling. *Neurocomputing*.

[28] Zenglin S., Yangdong Y., Wu Y. (2016). Rank-based pooling for deep convolutional neural networks. *Neural Networks*.

[29] Wang S. H., Govindaraj V., Gorriz J. M., Zhang X., Zhang Y. D. (2021). Explainable diagnosis of secondary pulmonary tuberculosis by graph rank-based average pooling neural network. *Ambient Intelligence and Humanized Computing*.

[30] Zhang Y. D., Satapathy S. C., Wu D., Guttery D. S., Górriz J. M., Wang S. H. (2021). Improving ductal carcinoma in situ classification by convolutional neural network with exponential linear unit and rank-based weighted pooling. *Complex & Intelligent Systems*.

[31] Zhang Y. D., Pan C., Chen X., Wang F. (2018). Abnormal breast identification by nine-layer convolutional neural network with parametric rectified linear unit and rank-based stochastic pooling. *Journal of Computational Science*.

[32] Zhang Y. D., Jiang X., Wang S. H. (2022). Fingerspelling recognition by 12-layer CNN with stochastic pooling. *Mobile Networks and Applications*.

[33] Ke Z., Le C., Yao Y. (2020). A multivariate grey incidence model for different scale data based on spatial pyramid pooling. *Journal of Systems Engineering and Electronics*.

[34] Chang J., Zhang L., Gu N. (2019). A mix-pooling CNN architecture with FCRF for brain tumor segmentation. *Journal of Visual Communication and Image Representation*.

[35] Skourt B. A., Hassani A. E., Majda A. (2021). Mixed pooling dropout for CNN regularization. *Journal of King Saud University Computer and Information Sciences*.

[36] Wang Y., Xiaoning S., Wu X. (2020). Two-stream face spoofing detection network combined with hybrid pooling. *Journal of Image and Graphics*.

[37] Qi B., Han Z., Kun Q. (2021). Multi-scale stacking attention pooling for remote sensing scene classification. *Neurocomputing*.

